# Atlas and Epitome of Operative Surgery

**Published:** 1899-06

**Authors:** 


ATLAS AND EPITOME OF OPERATIVE SURGERY. By Dr. O. Zücker-kandl, Privat Docent in the University of Vienna. Authorized translation from the German. Edited by James Chalmers Da Costa, M. D., Clinical Professor of Surgery in Jefferson Medical College, Philadelphia, etc., with 24 colored plates and 217 illustrations in the text. Published by W. B. Saunders, Philadelphia, Pa., 1898. Pages, 395. Price, cloth, $3.00, net.

Another volume of the beautiful hand-atlas series being published by Mr. Saunders. It is particularly directed to students, hence those operations that are usually demonstrated on the dead subject have received the greater share of attention; but the general surgeon will find much of interest and value that is new, and will, no doubt, have many of his old lessons vividly brought to mind again by the excellent colored plates and illustrations in black. As has already several times been said in passing upon other volumes of this series, these plates must be seen and studied before their beauty and true worth can be appreciated. Any library, no matter how full, will be incomplete without this series.

